# A microfluidic platform for in situ investigation of biofilm formation and its treatment under controlled conditions

**DOI:** 10.1186/s12951-020-00724-0

**Published:** 2020-11-11

**Authors:** Hervé Straub, Leo Eberl, Manfred Zinn, René M. Rossi, Katharina Maniura-Weber, Qun Ren

**Affiliations:** 1 Laboratory for Biointerfaces, Empa, Swiss Federal Laboratories for Materials Science and Technology, 9014 St. Gallen, Switzerland; 2 Department of Plant and Microbial Biology, University of Zürich, 8008 Zürich, Switzerland; 3 Institute of Life Technologies, University of Applied Sciences and Arts Western Switzerland (HES-SO Valais-Wallis), Sion, Switzerland; 4 Laboratory for Biomimetic Membranes and Textiles, Empa, Swiss Federal Laboratories for Materials Science and Technology, 9014 St. Gallen, Switzerland

**Keywords:** Microfluidics, Bacterial adhesion, Biofilm, Single-cell resolution, In situ analysis, Growth medium, Antimicrobial efficacy

## Abstract

**Background:**

Studying bacterial adhesion and early biofilm development is crucial for understanding the physiology of sessile bacteria and forms the basis for the development of novel antimicrobial biomaterials. Microfluidics technologies can be applied in such studies since they permit dynamic real-time analysis and a more precise control of relevant parameters compared to traditional static and flow chamber assays. In this work, we aimed to establish a microfluidic platform that permits real-time observation of bacterial adhesion and biofilm formation under precisely controlled homogeneous laminar flow conditions.

**Results:**

Using *Escherichia coli* as the model bacterial strain, a microfluidic platform was developed to overcome several limitations of conventional microfluidics such as the lack of spatial control over bacterial colonization and allow label-free observation of bacterial proliferation at single-cell resolution. This platform was applied to demonstrate the influence of culture media on bacterial colonization and the consequent eradication of sessile bacteria by antibiotic. As expected, the nutrient-poor medium (modified M9 minimal medium) was found to promote bacterial adhesion and to enable a higher adhesion rate compared to the nutrient-rich medium (tryptic soy broth rich medium ). However, in rich medium the adhered cells colonized the glass surface faster than those in poor medium under otherwise identical conditions. For the first time, this effect was demonstrated to be caused by a higher retention of newly generated bacteria in the rich medium, rather than faster growth especially during the initial adhesion phase. These results also indicate that higher adhesion rate does not necessarily lead to faster biofilm formation. Antibiotic treatment of sessile bacteria with colistin was further monitored by fluorescence microscopy at single-cell resolution, allowing in situ analysis of killing efficacy of antimicrobials.

**Conclusion:**

The platform established here represents a powerful and versatile tool for studying environmental effects such as medium composition on bacterial adhesion and biofilm formation. Our microfluidic setup shows great potential for the in vitro assessment of new antimicrobials and antifouling agents under flow conditions.
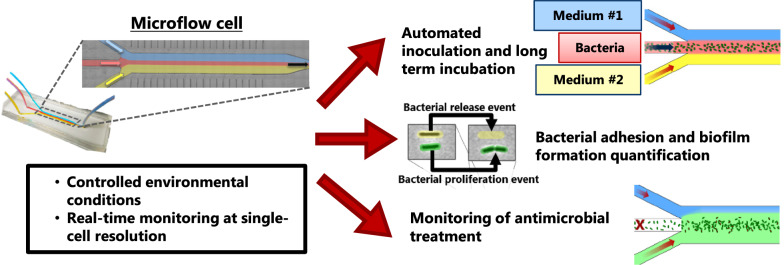

## Background

Microbial infections are a subject of major and growing concerns in the medical field, especially due to the dramatic increase of strains that are resistant to the currently available antibiotics. Considering 60–80% of all infections are complicated by involvement of biofilms [1], the biofilm mode of growth represents a major risk factor for the spread of antibiotic resistance [2]. Biofilm formation occurs in ordered series of stages, with initial reversible attachment of cells progressing into permanent adherence as bacteria multiply. Once firmly attached, the adherent bacteria alter their behavior, modulate their gene expression, and start producing a protective matrix of extracellular polymeric substance (EPS), which encases the emerging microcolony [3].

Traditionally, static, semi-static, and flow chamber assays have been used to investigate the influence of material properties and environmental factors on bacterial adhesion. For example, a microtiter plate-based assay has been reported as an accurate and convenient method for quantification of bacterial adhesion on textiles [4] and flow chambers have been employed to quantifying biomass formation under controlled flow condition [5]. Although these assays offer many advantages, their nature often does not allow real-time monitoring of bacterial adhesion and biofilm formation. Instead, they mostly rely on endpoint indirect measurements such as colony forming unit (CFU) counting or biomass quantification, which reduce the entire adhesion and growth processes to a single measurement. Microscopy-based real-time monitoring of bacterial adhesion is therefore highly desirable as it enables a deeper understanding of these processes.

Microfluidic systems have been gaining importance in the study of bacterial adhesion and biofilm formation. Not only do they enable real-time observation of the bacterial behavior but also they allow precise control over a broad range of environmental parameters such as hydrodynamic shear stress experienced by adherent bacteria and flux of nutrient as well [6, 7]. Furthermore, they offer aspect of in vivo relevance, given that bacteria are often subjected to flow in their natural environment.

Several studies have employed microfluidic approaches to investigate the influence of flow shear stress [8, 9], antimicrobial treatment [10, 11], and other environmental parameters [12] on bacterial adhesion and biofilm formation. One limitation of these approaches was the lack of spatial control over bacterial adhesion inside the chips. Lee et al. reported that bacteria unavoidably accumulated in the flow inlet area and close to the sidewall of the channels because of the lower flow and thus reduced shear stress at these sites [11].

Recently, Aznaveh et al. developed a sophisticated microfluidic flow chamber, which allowed to confine biofilm growth in a channel using flow-templating [13]. The same group further refined this technique to prevent upstream bacterial aggregation by using channels of varying width [14]. Using this flow-templating system, they were able to grow bacterial biofilms for several days under constant perfusion. However, microscopic observation of the bacteria was limited due to low magnification, as the design of the chip prevented close proximity between the specimen and the microscope objective.

Based on the knowledge provided by these studies, we aimed at developing a versatile, yet simple microfluidic system that allows real-time monitoring of bacterial adhesion and biofilm formation in a microflow cell (µFC) by high-resolution optical microscopy. The platform was designed in order to steer bacterial adhesion through flow focusing under homogenous hydrodynamic flow shear stress and to maintain constant feeding flow rates for more than 70 h with sterile medium.

Through this platform, we quantified the influence of two different media on adhesion kinetics of the medically relevant indicator strain *Escherichia coli* and the subsequent biofilm formation on a glass surface with high temporal and spatial resolution. The nutrient-poor medium was found to enable a higher adhesion rate compared to the nutrient-rich medium, however, the rich medium allowed faster colonization of the adhered cells than the poor medium under otherwise identical conditions. We revealed for the first time that this effect is caused by a higher retention of newly generated bacteria in the rich medium, rather than faster growth. Moreover, we used the platform to visualize in real time the killing activity of an antibiotic (colistin) treatment at the single-cell level, demonstrating the versatility of the platform. The novelty of this work lies in the established microfluidics platform capable of precise spatial control over bacterial colonization and single-cells level resolution, and by exploiting this platform we provided experimental evidence that bacterial initial adhesion rate does not necessarily correlate with subsequent biofilm formation rate.

## Results and discussion

### Design and operation of the microfluidic platform

We first tested a simple microfluidic chip containing a straight 100 µm wide channel made out of polydimethylsiloxane (PDMS) bonded on glass. Unsurprisingly, inhomogeneous bacterial adhesion and formation of large bacterial agglomerates along the chip sidewalls were observed (Fig. S1, Additional file 1). In general, a high density of bacteria was found close to the access holes as well as to the sidewalls of the channels due to the lower flow shear at these positions, similar to what has been reported before [11]. Furthermore, formation of so-called streamers was also observed after 3 h of continuous inoculum injection into the microchannel (Fig. S2, Additional file 1 and Video, Additional file 2).

The investigated *Escherichia coli* not only adhered to the glass surface but also to each other, leading to the formation of large clumps of cells in the area of low flow rates. This phenomenon has been reported previously [15, 16]. During our observations, the bacterial clumps were eventually washed away and thereby not only removed bacteria from the channel floor but also clogged the microchannel as has been reported by others [17]. This observation can be explained by the narrowing of the flow channel due to biofilm formation resulting in a local increase of the flow speed, which in turn increases the shear force. Along with the change of the flow profile the chance of removal of adhered cells is significantly increased.

**Fig. 1:**
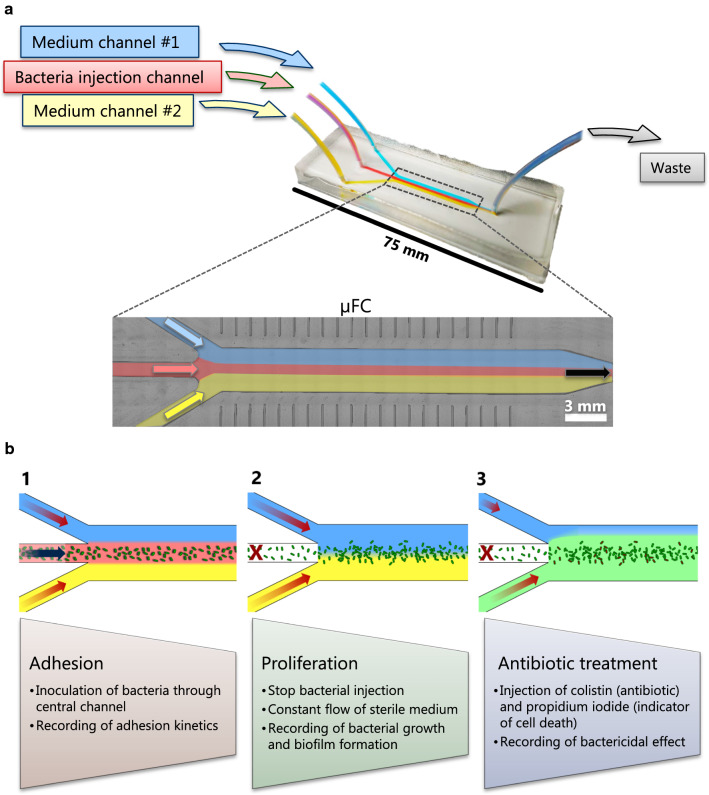
Design of the microfluidic platform with a close-up view of the µFC (**a**) and overview of its application example (**b**). The µFC is 180 µm high, 3 mm wide and 25 mm long

In order to overcome these limitations, we designed a tailor-made microfluidic µFC. The design includes three inlet channels that merge into a single chamber followed by an outlet channel (Fig. 1a). This arrangement enabled control of the flow rate from three different liquid reservoirs and thereby allowed spatially separated flows of different media in the same µFC chamber because of the laminar flow regime (Fig. S3, Additional file 1).

The flow-focusing principle was previously shown to be a reliable way to steer bacterial adhesion to the center of a flow chamber, effectively preventing bacterial adhesion on sidewalls [13, 18]. However, the design of the flow chamber restricted microscopic observation to low magnification. Indeed, the architecture of the flow chamber prevented the microscope objective from coming into close proximity with the specimen, therefore only allowing the use of low magnification objectives with long focal distances. With our novel microfluidic platform, we quantified the influence of growth medium on the kinetics of bacterial adhesion and biofilm formation at single-cell resolution under flow, which would have not been possible with the so-far reported systems.

The newly designed µFC was mounted on the stage of an inverted microscope so that the adherent bacteria on the bottom glass surface of the µFC could be imaged by wide-field microscopy. A bacterial suspension was injected in the central channel while medium was flown from the two outer channels, thus restricting the bacteria to the center of the µFC (Fig. 1b: adhesion phase). Since non-adhered bacteria cells were removed by the fluid motion, only adhered bacteria were visible. After a given period of time, the flow of inoculum in the central channel was stopped while perfusion of sterile medium from both outer channels was continued for up to 65 h in order to allow biofilm growth (Fig. 1b: proliferation phase). During this phase, images were recorded at several defined locations in the µFC. The recorded images were then processed by automated single-cell tracking analysis in order to measure surface coverage and the behavior of adherent cells.

Finally, the platform was used to investigate the antimicrobial effect of colistin by injecting the antibiotic into one of the inlet channels (Fig. 1b: antibiotic treatment).

### Automated and spatially controlled inoculation in the microflow cell

**Fig. 2:**
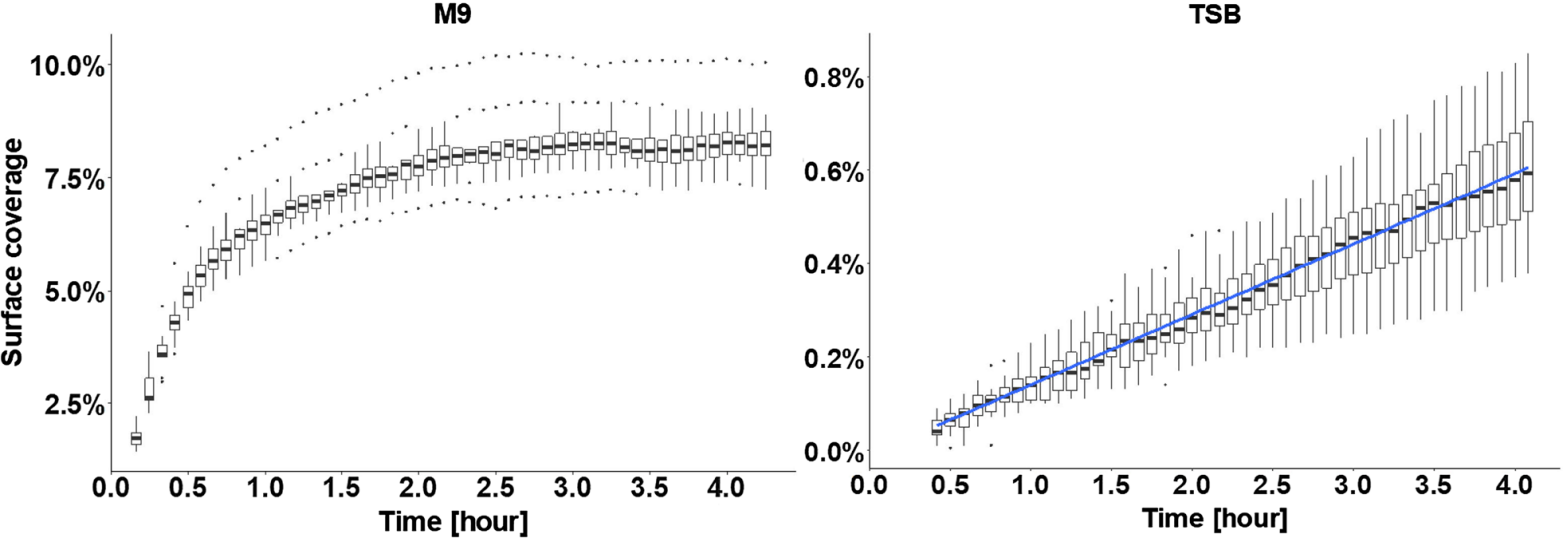
Influence of medium composition on *E. coli* adhesion in TSB and M9. Each boxplot represents the distribution of surface coverage at a given time point based on ten pictures acquired at different positions in the µFC. A linear regression model is fitted for the results in TSB (blue line, R^2^ = 0.77)

Utilizing the thus improved novel platform, we investigated the effects of medium composition on the initial attachment of *E. coli* and subsequent proliferation on the glass surface. Cells either cultured in rich medium tryptic soy broth (TSB) or modified minimal medium M9 (later simply referred as M9) were injected into the microfluidic chip. The cells that were in their exponential growth phase in TSB and M9 had an OD600nm of 0.30 and 0.26, respectively, measured just before inoculation. Bacteria on the glass surface were exposed to a maximal hydrodynamic flow shear rate of 412 s^−1^ with a Reynolds number of 4.7, which lies well within the laminar flow regime. M9 medium led to much faster cell adhesion and higher numbers of adhered bacteria than TSB medium within the same time period (Fig. 2), despite the fact that the M9 suspension contained a lower number of bacteria than the TSB suspension. After 0.5 h, cells suspended in M9 led to 5% surface coverage, whereas those in TSB barely reached 0.1%. Prolonged perfusion of 4 h resulted in the median surface coverage of 8 ± 0.7% for bacteria suspended in M9, and below 1% for those in TSB medium. The adhesion profile of bacteria in TSB was linear with a constant adhesion rate over the 4 h inoculation phase, whereas the adhesion rate of bacteria in M9 quickly decreased to reach a plateau after about 2.5 h of injection (Fig. 2). This saturation phenomenon could be due to the steric influence of adherent bacteria on the flow profile near the surface by preventing flowing bacteria from interacting with the glass surface, and thus hindering further colonization of the surface. On the other hand, since *E. coli* has been reported to exhibit a negative zeta potential [19], electrostatic repulsion from the adherent bacteria could prevent as well the new bacteria from interacting with the surface.

It has been reported that nutrient limitation and thus medium composition can play an important role in bacterial cell membrane composition and extracellular polymeric substance (EPS) production [20, 21] where starving bacterial cells were shown to have altered levels of sugars and proteins on their surface. Bacterial adhesion and biofilm formation are dependent on experimental conditions (e.g. media and material surface characteristics) and studied strains [22, 23]. Moreover, high level of adhesion of a given organism in a given medium does not imply high amount of biofilm formation [24]. Nevertheless, one can hypothesize that the reduced amount of nitrogen present in M9 medium compared to TSB could lead to different cell membrane composition and EPS production level and be the reason behind the different bacterial adhesion behavior that we observed.

Furthermore, the two different media used are likely to result in different conditioning films on the glass surface, which in turn can result in different physicochemical affinity between bacteria and glass surfaces [25], offering another explanation for the differences in adhesion. In order to test the latter hypothesis, water contact angle measurements were performed on glass slide pretreated with TSB, M9 medium or phosphate buffered saline (PBS) (Fig. S4, Additional file 1). TSB significantly increased the hydrophobicity of the glass surface compared to glass pre-treated with PBS and M9 (43° ± 11°, 20° ± 3°, and 17° ± 3°, respectively). This increase could be explained by the higher concentration of amino acids in TSB compared to that in M9 that can be readily adsorbed on the glass surface. The altered surface hydrophobicity could result in lower bacterial adhesion in TSB due to less favorable physicochemical affinity between bacteria and glass.

Broadly speaking, these results highlight the importance of medium selection when conducting adhesion assays and when comparing results obtained with different media. Moreover, biofilms occurring on medical devices such as catheters are formed in a different nutritional environment than standard media used in the laboratory [26]. It is thus necessary to employ media that mimic the actual in vivo environment in order to aim at predictive conditions for in vitro studies.

### Automated single-cell tracking to monitor surface colonization by bacteria

The independent flow control of the three channels and the laminar flow regime enabled us to use two different media (TSB and M9, respectively) synchronously in one experiment. Hence, the influence of two different media on biofilm formation could be investigated within a single µFC with otherwise identical conditions. Moreover, the flow-focusing design conveniently allowed us to use dedicated channels to inject an *E. coli* inoculum and to deliver sterile medium for extended periods of time to the adhered bacteria in the µFC, thus eliminating the undesirable effects of bacterial growth in the feeding channels, such as clogging and medium alteration.

**Fig. 3:**
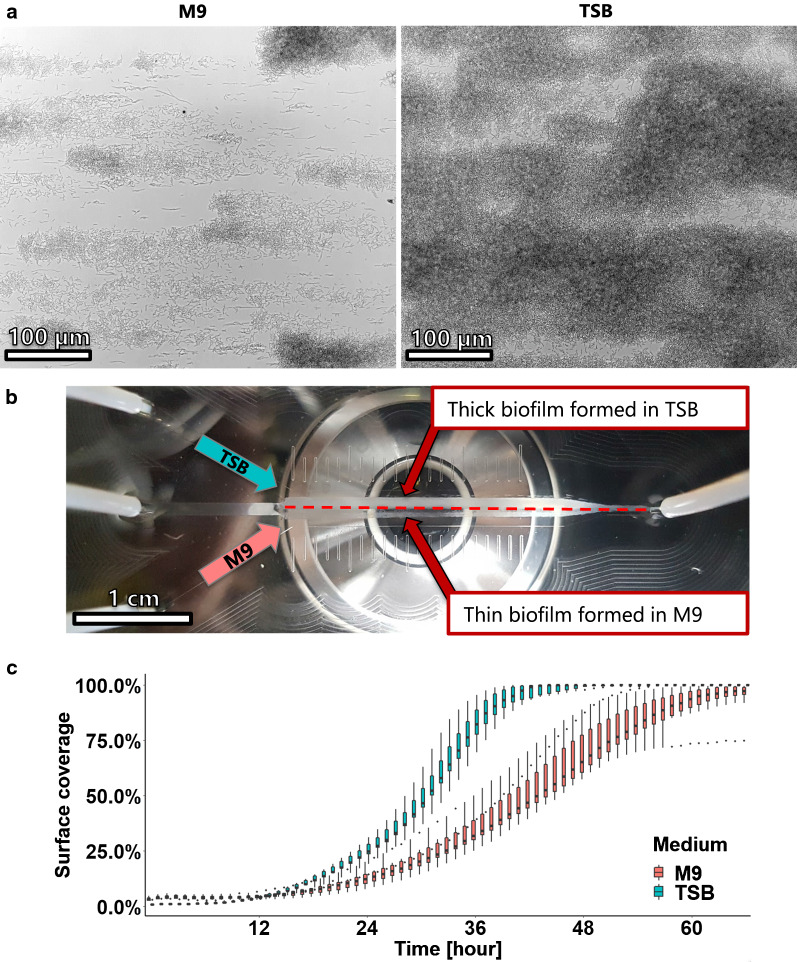
Influence of medium composition on biofilm formation. Bacteria in PBS were injected in the center of the channel over the course of one hour. The chamber was subsequently perfused with TSB medium and M9 medium simultaneously during 66 h. **a** Micrographs of the biofilm formed after 36 h of incubation show the different rates of biofilm formation. **b** Overview of the biofilm formed after 66 h, the difference in biofilm thickness due to the different medium compositions can be clearly seen. **c** Biofilm growth was assessed by quantifying the increase of surface coverage with time. Each box-plot represents the distribution of surface coverage based on ten images for each medium composition every hour

TSB was injected into the left outer channel and M9 into the right outer channel so that the two sides of the µFC were in contact with either of the media (Fig. S5, Additional file 1). Bacteria were suspended in PBS and injected through the central channel of the observation chamber for one hour. Thereafter, microscopic images of the growing biofilm were taken at defined locations in the observation chamber either in the M9 perfused area, the TSB perfused area or at the interface region of the two media (Fig. 3a and Videos, Additional files 3–5). Bacterial growth is clearly visible under bright field time-lapse microscopy, namely bacterial elongation and binary fission, demonstrating the advantage of the established microfluidics platform in allowing analysis at single cell resolution. The biofilm that has developed after 66 h of incubation is shown in Fig. 3b. The two different perfusion regions can be easily discriminated. The biofilm formed in TSB became much more opaque than the one that was incubated in M9 medium, indicating that TSB promoted the formation of a thicker biofilm.

The surface coverage was used to quantify the biofilm formation as it reflected the increase of biomass on the glass surface. This analysis showed a fast colonization of the surface by bacteria grown in TSB (Fig. 3c); after 40 h the observed region were almost completely covered. By contrast, bacteria grown in M9 medium colonized the surface more slowly and some regions remained uncovered even after 66 h of incubation. At first sight, this data is in agreement with results obtained from the semi-static assay, where more biofilm was formed in TSB than in M9 (Fig. S6, Additional file 1). In addition, planktonic *E. coli* were also found to grow more slowly and reached a lower cell density in M9 than in TSB (Fig. S7, Additional file 1). This can be explained by the readily available nutrients such as amino acids in TSB medium. However, unlike growth in static assay where the amount of nutrient is limited by the volume of liquid, bacteria grown in the µFC are constantly supplied with fresh medium. One should therefore expect that the nutrient concentration cannot be the limiting factor for the growth of *E. coli* cells in M9, at least during early phase of biofilm formation.

**Fig. 4:**
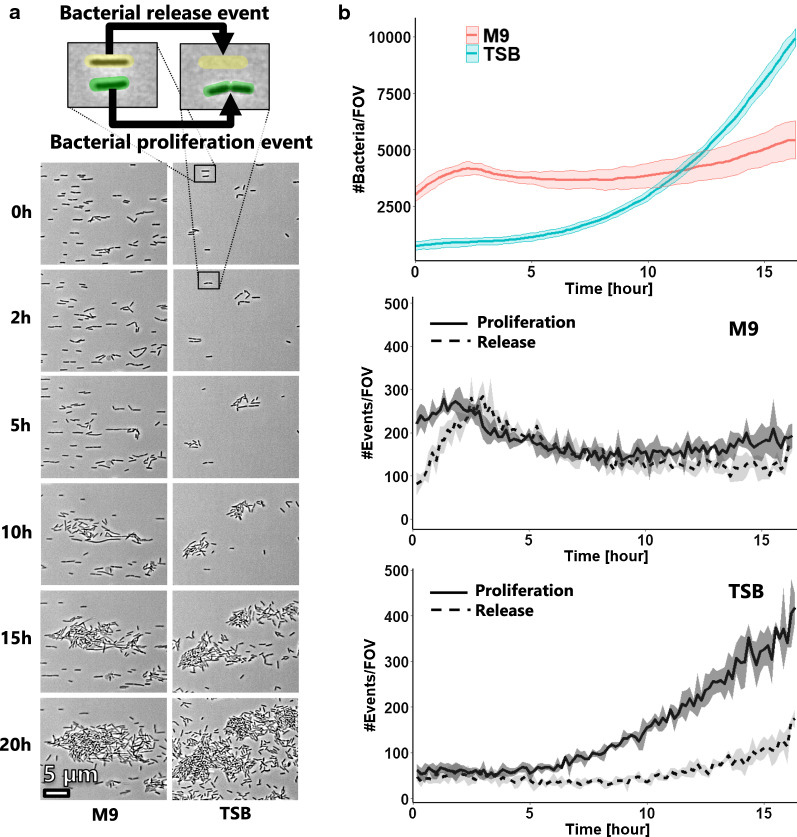
Single-cell tracking analysis of early biofilm formation. **a** Image sample of the adherent bacteria during the early stage of biofilm formation in M9 and TSB medium. **b** Single-cell tracking was performed to quantify the amount of adherent bacteria, the generation of new ones, and their release from the surface. The data is plotted as the mean count of bacteria and events per field of view (FOV) (lines) with standard deviation (shaded area) based on three locations with a sampling rate of 10 min

To gain better understanding on the observed colonization profile, we performed single-cell tracking analysis during early stage of biofilm formation in order to monitor the behavior of cells on the surface. This enabled us to precisely follow the dynamics of the bacterial colonization on the surface by quantifying the bacterial proliferation and the bacteria being released in the flowing liquid (Fig. 4a).

The evolution of cell numbers with time in both media can be seen in Fig. 4b. More cells were originally present on the glass surface in contact with M9 than with TSB and their rate of growth was faster for the first 2.5 h of incubation. It then stagnated, while the growth rate of bacteria in TSB gradually increased. Bacteria growing in TSB eventually overtook those growing in M9 after 12 h of incubation.

Single-cell tracking enabled us to not only record the amount of bacteria on the surface at a given time but also to follow the generation of new bacteria (through cellular division) and the release of bacteria from the surface (Fig. 4b). We observed that bacteria actively divided at the beginning of the incubation in both media. Surprisingly, bacteria proliferated faster in M9 than in TSB for more than 10 h. However, more bacteria in M9 were released from the surface than in TSB. An equilibrium between generation and release was observed between 2.5 h and around 10 h, explaining the lag in surface colonization in M9. This phenomenon was not observed for cells exposed to TBS: after 5 h of incubation, progressively more bacteria were generated on the surface than left, resulting in an exponential colonization phase.

To summarize, the lag in population growth on the glass surface in M9 is not due to delay or slower bacterial growth but rather caused by the release of bacteria into the liquid. These differences of cell growth dynamics between the two media were visible on the pictures as bacteria growing in TSB formed clusters early on while bacteria growing in M9 stayed isolated on the surface for longer periods of time (Fig. 4a).

These discoveries highlight the importance of a suitable tool like the established microfluidic platform here to allow detailed investigations at single-cell level, and the medium selection for the design of relevant biofilm models. A major advantage of our platform is the fact that any planktonic bacteria released from the growing biofilm are readily cleared from the µFC thanks to constant medium perfusion. By contrast, planktonic cells have an unavoidable influence in biofilm studies performed under static condition, in which careful rinsing steps have to be involved for the removal of planktonic cells before quantification or further processing and analysis of the biofilm. Moreover, planktonic bacteria can compete with sessile cells for nutrients during growth of the biofilm. By ensuring perfusion of sterile medium inside the µFC and clearing away planktonic cells, the platform overcomes this issue and permits the study of sessile bacteria alone in a controlled environment.

Furthermore, the homogenous biofilm growth along the entire length of the µFC (Fig. 3b) indicated that the medium flow rate was sufficient to provide enough nutrients to the biofilm formed on the whole length of the µFC. The applied flow rate of 400 µl/min resulted in a mean flow velocity of 12 mm/s in the µFC and thus a residence time of approximately 2 s for the medium within the µFC (dilution rate of 30 min^−1^). It is thus highly likely that sufficient nutrient in the flow is provided to obtain homogenous biofilm, even at the end of the µFC. In addition, it can be speculated that the flow of oxygen was sufficient for aerobic growth condition.

### Visualization and quantification of antibiotic activity

**Fig. 5:**
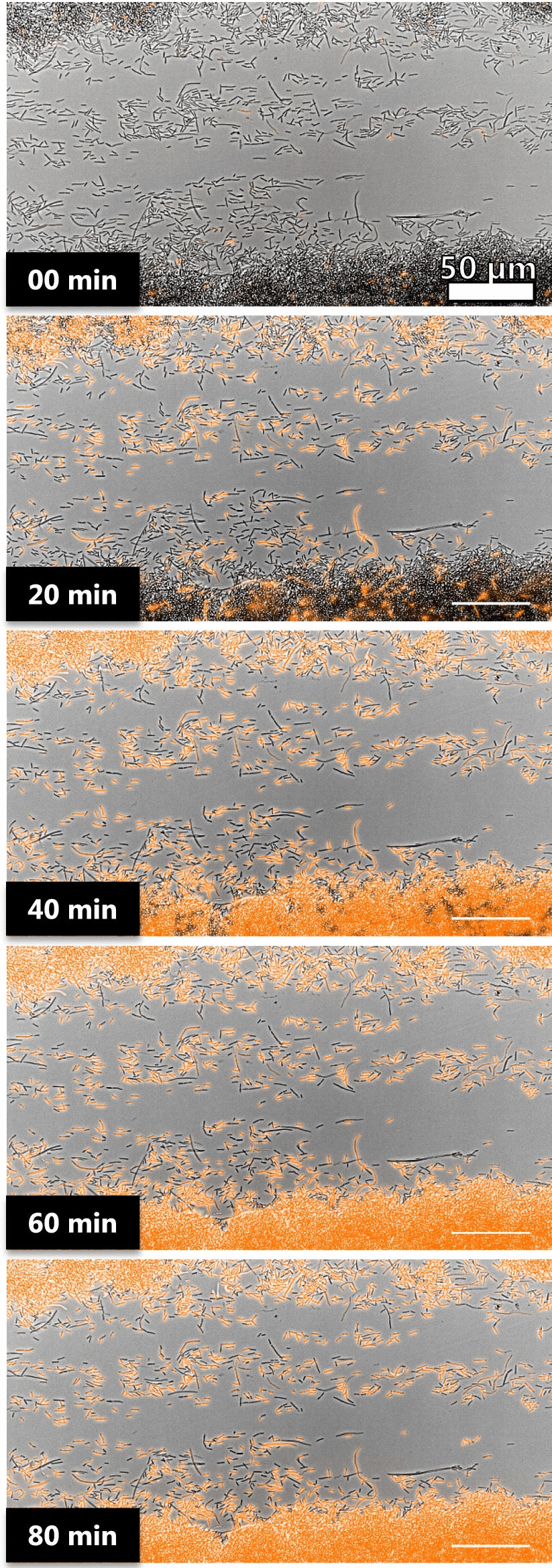
Time-lapse microscopy of adherent bacteria treated with 2 µg/ml of colistin and PI. Images were acquired every 30 s for 80 min. PI signal (in orange) was overlaid with bright-field images. The killing effect of colistin can be recorded down to the single cell level

To demonstrate the versatility of the developed platform, *E. coli* biofilms were treated with colistin, an effective antibiotic against Gram-negative bacteria, and the killing action was followed in real-time by using a method described by Avalos Vizcarra et al. [27]. Briefly, propidium iodide (PI) was added to the antibiotic solution at a non-cytotoxic concentration. PI can penetrate membrane-deficient cells and confer to these (dead) cells a bright red fluorescent signal. By following the emergence of the red fluorescent signal, colistin was found to rapidly kill bacteria and after 80 min of injection, only a small fraction of the cells were still alive (Fig. 5 and Video, Additional file 6).

The platform established here can be utilized for screening and assessment of novel antimicrobial agents against surface-associated bacteria and biofilms. Unlike traditional assays that rely on indirect measurements of cell viability either by optical density or colony counting, our assay allows direct visualization of single live/dead cells and reveals in-depth information about the performance of an antimicrobial agent. Moreover, the platform is fully compatible with confocal microscopy and high magnification with oil immersion objectives making the recording of spatial-resolved antibiotic activity on biofilm possible.

### Stability and versatility of the platform

The platform presented here is extremely versatile and can be customized to suit a broad range of applications such as investigating the biofilm formation ability of different bacteria including mutants under defined and controlled conditions or the effect of a molecule of interest on adherent bacteria to name a few. Besides, different flow shear stresses can be easily applied by adjusting the flow rate and the dimensions of the microchannels, and different media can be perfused synchronously.

Gas bubbles are a common burden in most microfluidic applications, as they can severely alter the flow characteristics. The bubbles can spontaneously form inside a microfluidic system by degassing out of liquid phase. The likelihood of this phenomenon increases with prolonged operation time and long-term incubation is therefore more prone to bubble formation than short-term experiments. By using a pressure driven flow and working with a positive pressure in the range of 500 mbar, the spontaneous occurrence of bubbles was avoided without the use of a bubble trapping system, thus keeping the complexity of the platform low. The pressure-driven flow also enabled the use of conventional glass bottles as medium reservoir hence allowing large volumes to be perfused, which would have been impossible with a traditional syringe pump [28]. Moreover, using a pressure controller drastically reduced the risk of leakage in case of clogging of the system by preventing the rise of pressure that can occur with traditional pumps. By using flow sensors, pressure applied to the reservoir could be continuously adjusted in order to maintain constant flow rate throughout the whole experiment.

Additionally, the relatively large dimension of the microfluidic system (channel width ranging from 1 to 3 mm) and the absence of intricate channel geometry made it possible to manufacture the mold used for chip fabrication by computer numerical control (CNC) milling instead of photolithography, which greatly reduces the cost of production. Moreover, stereolithography is another cost-effective manufacturing method also suitable for mold fabrication and gaining popularity [29].

Here we employed our platform to investigate the influence of medium composition on bacterial adhesion and biofilm formation under constant flow rate. This study demonstrated that the platform is suitable for studying the dependence of environmental parameters (nutrition, shear stress) on the described phenomena as well as for testing novel antimicrobial agents. Firstly, their performance can be assessed on adherent bacteria in a more clinically relevant setting compared to traditional assays. Secondly, real-time monitoring of the activity of an antimicrobial agent at the single cell level will allow a deeper understanding of its mode of action.

## Conclusion

The present study designed and established a novel microfluidic system for investigating bacterial adhesion on surfaces and biofilm growth in a µFC under various and well-controlled conditions. Spatially controlled and homogeneous bacterial adhesion was achieved on the floor of the µFC. Using the established platform, it was found that more adherent bacteria in nutrient-poor medium did not lead to higher surface colonization and biofilm formation. The reason was demonstrated to be the higher tendency for bacteria growing in M9 to leave the surface compared to those growing in TBS. This analysis was achieved by using automated single-cell tracking powered by the microfluidics platform.

The results of the study demonstrated that the newly designed microfluidic platform can be exploited for the study of bacterial and surface interactions as well as antimicrobial performance without requiring advanced or expensive equipment. Moreover, the established platform represents an exceptional tool for studying the in situ activity of antimicrobial agents against surface-associated bacteria and cells in a biofilm. Finally, the platform enables the study of a wide range of organisms and growth conditions.

## Materials and methods

### Chemicals and reagents

Chemicals and reagents used in this study were purchased from Sigma Aldrich (Switzerland) if not mentioned otherwise.

### Bacterial strains and cultivation conditions

*E. coli* DH5α were plated on tryptic soy agar and grown at 37 °C overnight. A single colony was picked and used as inoculum for overnight liquid culture. Overnight liquid cultures were grown in tryptic soy broth (TSB) or modified M9 minimal medium (M9) [30] at 37 °C and 160 rpm.

### Fabrication of the microfluidic device and fluidic setup

The design of the microfluidic chip was derived from commercially available 3in1 µ-slide (ibidi, Germany). The microfluidics chips were fabricated using standard soft lithography technique. Briefly, a poly(methyl methacrylate) (PMMA) mold was produced by CNC machining. PDMS (Sylgard 184 silicone elastomer kit, Dow Corning GmbH, Germany) was prepared to a weight ratio of 10:1 (base: curing agent), thoroughly mixed, degassed, poured over the PMMA mold, and cured at 60 °C overnight.

Access holes were perforated into the solidified PDMS stamps with a biopsy punch (1.7 mm diameter, Integra Miltex, USA). The stamps were then bonded on a glass slide (25 × 75 × 0.8 mm, Corning, USA) by low-pressure air plasma (Harrick Plasma, USA) and further incubated at 60 °C for 1 h.

Each chip contained three 1000 µm wide and 180 µm high rectangular inlet channels that merged into a 3000 µm wide and 180 µm high observation chamber. The observation chamber was 25 mm long and was terminated by a 1 mm wide outlet channel. Flow control was ensured by a 4-channel OB1 pressure controller and microfluidic flow sensors (Elveflow, France). The controller was used to pressurize 1 l glass bottles that were used as reservoir for medium perfusion on the two outer channels and 15 ml falcon tube used for bacterial suspension reservoir. Interconnection of the different flow components with the microfluidic chip was done using 1.6 mm polytetrafluoroethylene (PTFE) tubing (Bola, Germany).

### Microfluidic experimental workflow

To ensure that the bacteria were in the exponential growth phase during experiments, overnight liquid cultures were diluted to an OD600nm value of 0.1 in fresh TSB or modified M9 medium and incubated for 2 h at 37 °C and 160 rpm before use. For the experiments with dual medium composition, bacteria were grown overnight in TSB, diluted to 0.1 of OD600nm in fresh TSB and incubated for 2 h at 37 °C and 160 rpm, spun down at 4500 rpm for 15 min, resupsended in PBS and washed twice by repeating the centrifugation, and finally resuspended in PBS to an OD600nm value of 0.1 before use.

The microfluidic chips were imaged using a fully automated Nikon Ti2E inverted microscope fitted with a 40 × air objective, epifluorescent and diascopic (bright-field and phase contrast) illumination, and a digital camera. The preliminary experiments were performed on a Zeiss Axio Observer A1 inverted microscope fitted with a 40 × air objective.

All experiments were performed at room temperature (approximately 25 ºC).

The experimental workflow developed in this study is summarized in Fig. 1. Briefly, the entire system was sterilized with 70% ethanol. Then, 70% ethanol reservoirs on the two outer channels were carefully replaced with sterile growth medium in 1 l bottles in order to prevent entrance of air bubble in the system. Growth medium was flown into the system to flush the remaining ethanol out and to prime the observation chamber with medium. Next, a 15 ml falcon tube containing the bacterial suspension was connected to the central channel and injected into the observation chamber. Bacterial adhesion on the glass floor of the observation channel was recorder at 60 × magnification every 5 min. After the injection was completed, the flow of channel 3 was stopped by closing a manual valve so that only sterile medium from the two outer channels entered the observation chamber. Bacterial growth and biofilm formation were recorded every 10 min for up to 66 h. For the experiment with single medium, the injection phase was conducted for 4 h with a flow rate of 175 µl/min on the outer channels and 50 µl/min on the central channel. By contrast, for the experiments involving both media (M9 and TSB) at the same time and bacteria suspended in PBS, the injection phase lasted for 1 h with a flow rate of 25 µl/min on the two outer channels and 70 µl/min on the central channel in order to generate a wider seeding zone. After the bacterial suspension flow was shut down, the incubation phase was initiated, the flow of both medium channels was trimmed up to 200 µl/min and image acquisition was performed every 10 min at 40 × magnification for up to 66 h.

Antibiotic treatment was performed as follow: 10 ml of propidium iodide (PI) 2 µM mixed with colistin 2 µg/ml in PBS was prepared in a 15 ml Falcon tube and connected to one of the outer channels after having previously incubated bacteria in the system for 66 h with M9. The colistin/PI mixture was then injected into the chip. Images were recorded every 30 s with red fluorescence and bright-field to allow the observation of dying bacteria as the colistin compromised their membrane and allowed PI to enter the cytosol and to bind to DNA resulting in a strong red fluorescence. The treatment was performed for 90 min.

### Hydrodynamic shear stress and Reynolds number calculation

The shear rate γ in s^−1^ generated on the floor of the microchannels was calculated using the following formula [32]:

1 $$\gamma = \frac{6Q}{{wh^{2} }},$$

where *Q* is the volumetric flow rate, *w* is the channel width, and *h* is the channel height. This formula was derived for the case where *w* >  > *h* (parallel plate flow) assuming the medium is a Newtonian fluid.

The Reynolds number (Re) was calculated using the following formula:

2 $${\text{Re}} = \frac{dDv}{{\upmu }},$$

where *d* is the density of the fluid, *D* is the hydraulic diameter of the channel, *v* is the mean flow velocity and *µ* is the dynamic viscosity of the fluid. 1000 kg/m^3^, 340 µm, and 0.89 mPa s were used for d, D, and *µ*, respectively.

### Single-cell tracking analysis

Single-cell tracking was performed on a subset of the images recorded for surface coverage quantification (three randomly-selected locations from beginning of incubation to 16 h onward) with the software ImageJ [33] and the plugin TrackMate [34]. Briefly, bright-field images were first inverted and converted to 8-bit pixel depth. A LoG detector with a blob size of 2 µm was used in order to segment bacteria on each image. A simple LAP tracker with 5 µm max linking and gap-closing distances was then used for the tracking computation.

### Statistics

Data generated by Nikon Element software and ImageJ were parsed and plotted with R. The graphs were plotted either as mean with standard deviation or as standard boxplot; the lower and upper hinges (bottom and top of the rectangle) correspond to the first and third quartiles (the 25th and 75th percentiles) and are intersected by the median line. The upper whisker extends from the top hinge to the largest value no further than 1.5 × the distance between the first and third quartiles. Similarly, the lower whisker extends from the bottom hinge to the smallest value at most 1.5 × the distance between the first and third quartiles. Outlier data beyond the end of the whiskers were plotted individually.

## Supplementary information

**Additional file 1: Figure S1.** Bacterial adhesion profile in straight narrow microchannels. **Figure S2.** Bacteria streamer formation in microfluidic channel. **Figure S3.** Demonstration of the flow-focusing principle by visualization of the flow streamlines. **Figure S4.** Water contact angle measurement on glass slide pre-incubated with phosphate buffered saline (PBS), TSB, and M9 medium. **Figures S5.** Flow profile in the µFC visualized by fluorescence microscopy. **Figures S6.** *E. coli* biofilm formed in TSB and M9 media in semi-static condition. **Figures S7.** *E. coli* planktonic cell growth.

**Additional file 2: Video.** Time-lapse microscopy of bacterial streamer in narrow channel. The microchannel was perfused with bacteria in M9 medium at 2 µl/min. The video was taken after 3.5 h of perfusion and spans 7 min.

**Additional file 3: Video.** Time-lapse microscopy of Biofilm grown in M9.

**Additional file 4: Video.** Time-lapse microscopy of Biofilm grown in TSB.

**Additional file 5: Video.** Time-lapse microscopy of Biofilm grown with M9 and TSB simultaneously. The top half of the biofilm is in contact with TSB whereas the bottom half of the biofilm is in contact with M9 medium.

**Additional file 6: Video.** Time-lapse microscopy of colistin treatment on adhering bacteria.

## Data Availability

The datasets generated and analyzed during the current study are available from the corresponding author on reasonable request.

## Abbreviations

CV: Crystal violet
µFC: Micro flow cell
PDMS: Polydimethylsiloxane
PMMA: Poly(methyl methacrylate)
TSB: Tryptic soy broth
