# Supplementary material for: A microfluidic platform for in situ investigation of biofilm formation and its treatment under controlled conditions
Source: J Nanobiotechnology. 2020 Nov 11;18:166. doi: 10.1186/s12951-020-00724-0 (PMC7661213; doi:10.1186/s12951-020-00724-0)
Supplement: Supplementary file 1 — Additional file 1: Figure S1. Bacterial adhesion profile in straight narrow microchannels. Figure S2. Bacteria streamer formation in microfluidic channel. Figure S3. Demonstration of the flow-focusing principle by visualization of the flow streamlines. Figure S4. Water contact angle measurement on glass slide pre-incubated with phosphate buffered saline (PBS), TSB, and M9 medium. Figures S5. Flow profile in the µFC visualized by fluorescence microscopy. Figures S6. E. coli biofilm formed in TSB and M9 media in semi-static condition. Figures S7. E. coli planktonic cell growth. [file 12951_2020_724_MOESM1_ESM.docx]

Additional Information for

**A Microfluidic Platform for in situ Investigation of Biofilm Formation and its Treatment under Controlled Conditions**

**Hervé Straub^1, 2^, Leo Eberl^2^, Manfred Zinn^3^, René M. Rossi^4^, Katharina Maniura-Weber^1^, Qun Ren^1^***

^1^Laboratory for Biointerfaces, Empa, Swiss Federal Laboratories for Materials Science and Technology, CH-9014, St. Gallen, Switzerland

^2^Department of Plant and Microbial Biology, University of Zürich, CH-8008 Zürich, Switzerland

^3^Institute of Life Technologies, University of Applied Sciences and Arts Western Switzerland (HES-SO Valais-Wallis), Sion, Switzerland

^4^Laboratory for Biomimetic Membranes and Textiles, Empa, Swiss Federal Laboratories for Materials Science and Technology, CH-9014 St. Gallen, Switzerland

*** Correspondence:**[Qun.Ren@empa.ch](mailto:Qun.Ren@empa.ch)

# Supporting Figures


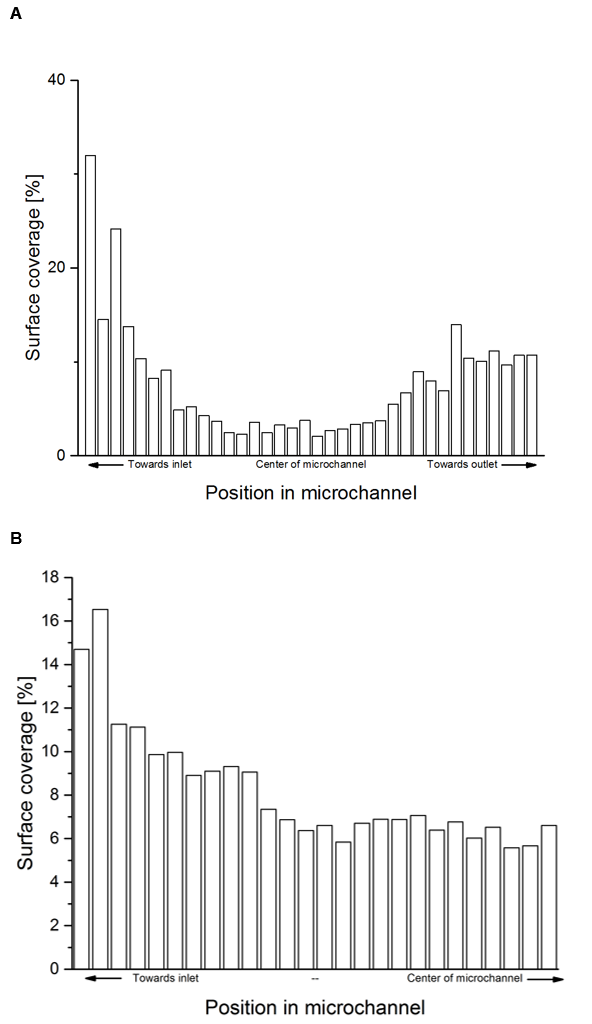


**Figure S1.** Bacterial adhesion profile in straight narrow microchannels. Two straight microchannels were perfused with a bacterial suspension in M9 for 3.5 h at 1 µl/min. Pictures along the microchannels were taken at regular interval to quantify surface coverage variation throughout the microchannel from inlet to outlet in one microchannel (**A**) and from inlet to center of the second microchannel (**B**). The field of view of every picture covered the whole width of the channels i.e. 100 µm width and 220 µm length**.**

**
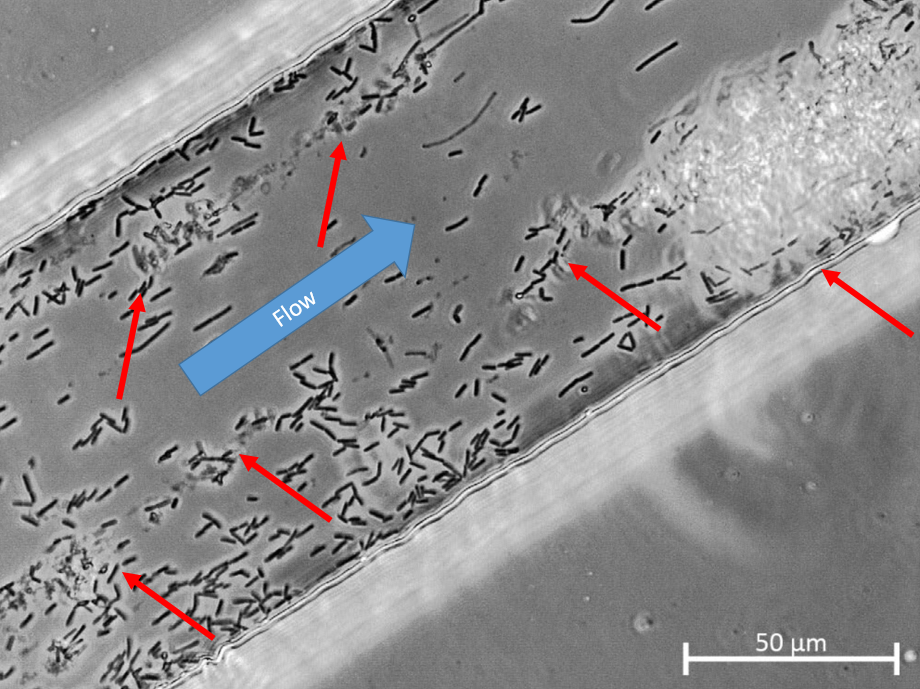
**

**Figure S2.** Bacteria streamer formation in microfluidic channel. Bacteria not only adhered to the glass floor but also to each other, leading to the formation of large bacteria clumps that were washed away through the channels (see Video, Additional file 2). Unlike motionless bacteria adhering to the glass floor of the channel, streamer can be seen undulating in the flow. Red arrows point to several parts of different streamers.


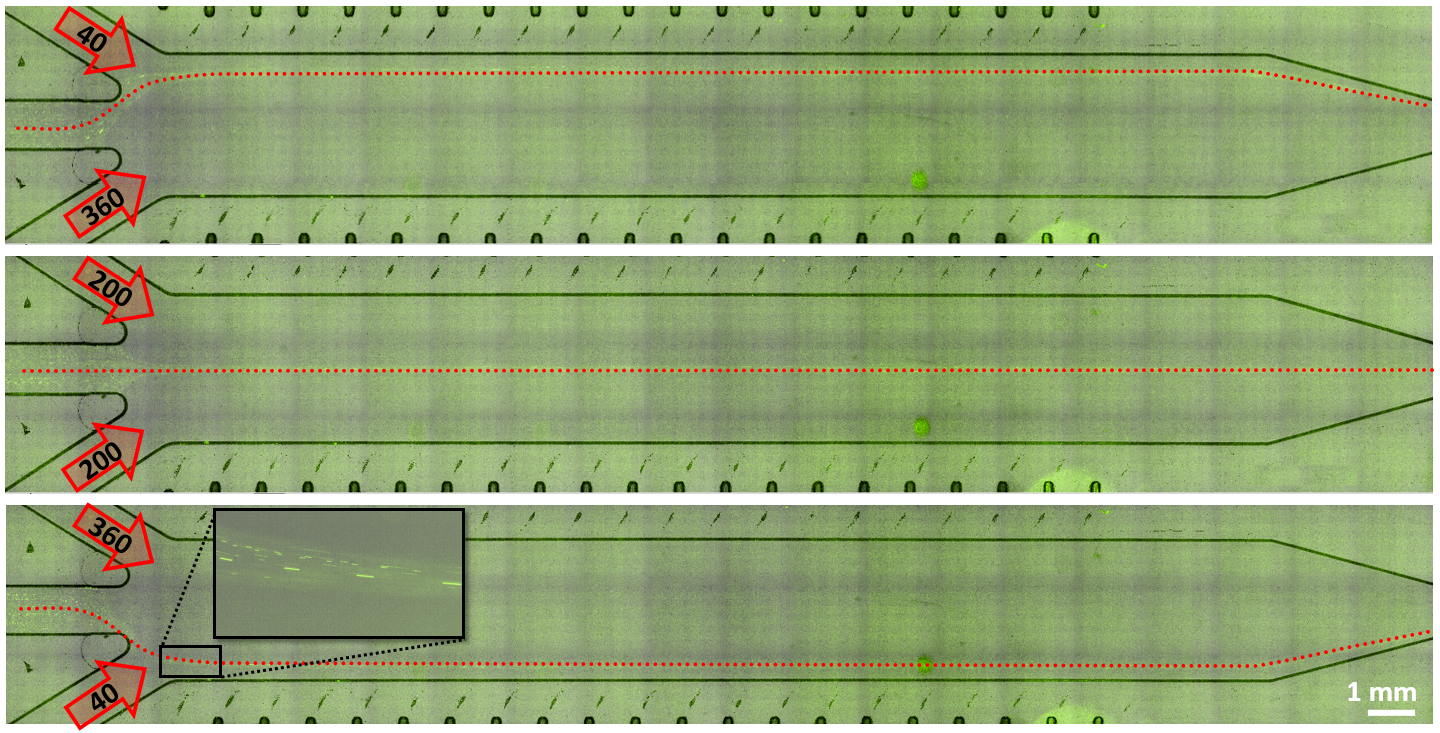


**Figure S3.** Demonstration of the flow-focusing principle by visualization of the flow streamlines (stitched bright field images with green fluorescence overlays at 10x magnification) under three different flow distributions. The three different streams effectively stay segregated as they flow through the microflow cell. The flow repartition can be spatially controlled by altering the respective flow value of the three inlet channels. The separation between the two flow streams coming from the two outer inlets was visualized by injecting a suspension of fluorescent polystyrene beads (diameter = 1µm) in the central inlet channel. A red dashed line is overlaid over the flow of fluorescent beads highlighting the streamlines. Values in the arrows indicate the respective flow rate of each inlet channel (in µl/min).

**Figure S4.** Water contact angle measurement on glass slide pre-incubated with phosphate buffered saline (PBS), TSB, and M9 medium. TSB pretreatment resulted in a statistically significant higher water contact angle (CA) than PBS and M9, whereas M9 did not significantly influence water contact angle compared to PBS (Student T-test, *p-*value < 0.001, n = 14)

**
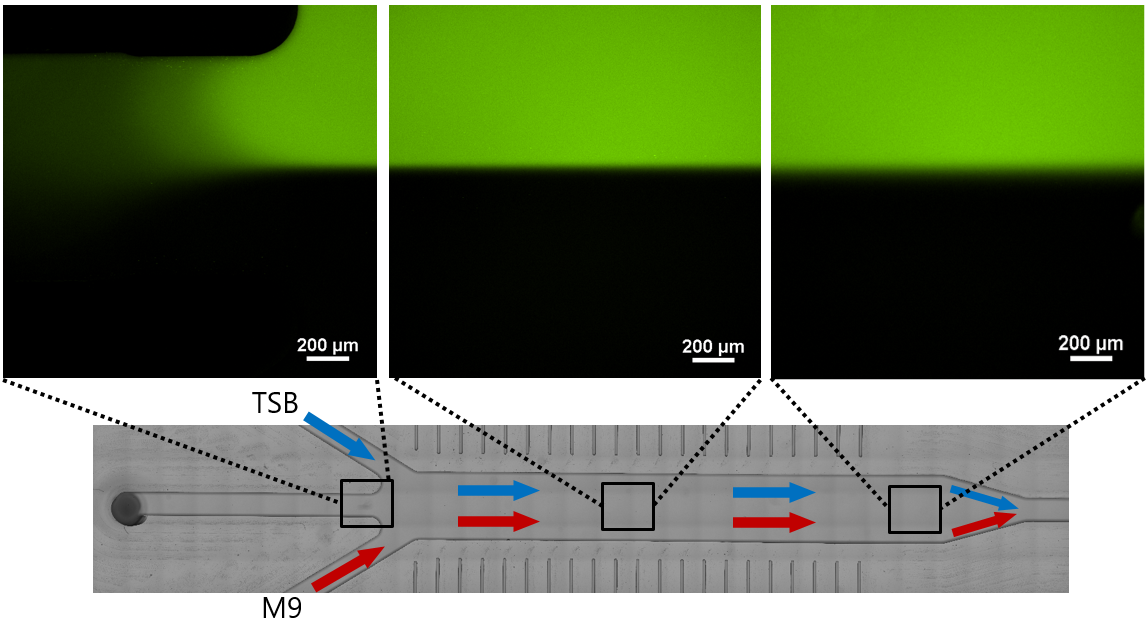
**

**Figure S5.** Flow profile in the µFC visualized by fluorescence microscopy. The laminar flow regime in the µFC is demonstrated by exploiting the higher autofluorescence property of TSB compared to that of M9. Fluorescence images acquired at 10x magnification at the entrance, middle and exit of the µFC showed that the two streams of medium (blue arrow for TSB with high fluorescent signal, red arrow for M9 with low fluorescent signal) only mix to a limited extent by diffusion in the µFC. The slightly blurrier interface between the two media at the exit of the µFC (image on the right) confirmed that molecular diffusion is negligible all along the µFC for the flow parameters that were used during biofilm growth.

## Figure S6. *E. coli* biofilm formed in TSB and M9 media in semi-static condition. Overnight liquid cultures were diluted to an optical density OD600nm value of 0.1 with TSB or M9. 200 µL of bacteria suspension per well were added to transparent flat bottom polystyrene 96-well plates (BRANDplates® pureGrade™). Plates were incubated for 24 hours at 37°C and 40 rpm. The biofilm formed in the wells was washed once with 350 µL 0.9% NaCl solution before quantification with crystal violet (CV) staining, as described previously (1). 250 µL 0.5% CV were added per well of the microplate. The plate was incubated for 30 min at 25°C before removing the staining solution, and then washed three times with 350 µL 0.9% NaCl solution. After removing the washing solution, 100 µL of 96% ethanol was added per well to dissolve the biofilm bound CV by gently knocking the plate. Absorbance was measured at 595 nm. Error bars represent 16 repeats in one experiment. The difference of biomass between the two growth conditions is statistically significate (T-test with *p*-value < 0.001)


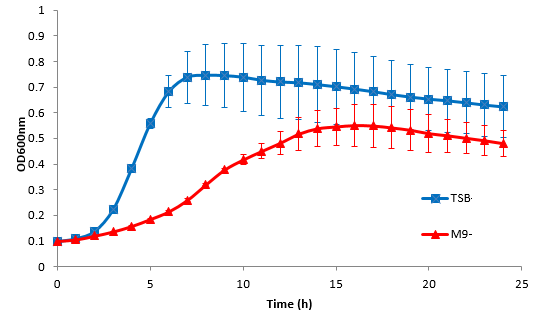


**Figure S7.** *E. coli* planktonic cell growth. *E. coli* DH5α was grown in TSB or M9 medium in a 96-well plate at 37°C and 40 rpm. The growth was followed every 0.5 h by measuring the optical density at 600 nm. Error bars represent 16 individual replicates done in one experiment.

1. Stiefel P, Rosenberg U, Schneider J, Mauerhofer S, Maniura-Weber K, Ren Q. Is biofilm removal properly assessed? Comparison of different quantification methods in a 96-well plate system. Appl Microbiol Biotechnol. 2016;100(9):4135–45.
